# Gene Therapy: The Next-Generation Therapeutics and Their Delivery Approaches for Neurological Disorders

**DOI:** 10.3389/fgeed.2022.899209

**Published:** 2022-06-22

**Authors:** Abhik Paul, Michael G. Collins, Hye Young Lee

**Affiliations:** Department of Cellular and Integrative Physiology, The University of Texas Health Science Center at San Antonio, San Antonio, TX, United States

**Keywords:** CRISPR/Cas9, gene therapy, *in vivo* gene editing, neurodevelopmental disorders, neurodegenerative diseases, therapeutics, viral vector, non-viral vector

## Abstract

Neurological conditions like neurodevelopmental disorders and neurodegenerative diseases are quite complex and often exceedingly difficult for patients. Most of these conditions are due to a mutation in a critical gene. There is no cure for the majority of these neurological conditions and the availability of disease-modifying therapeutics is quite rare. The lion’s share of the treatments that are available only provide symptomatic relief, as such, we are in desperate need of an effective therapeutic strategy for these conditions. Considering the current drug development landscape, gene therapy is giving us hope as one such effective therapeutic strategy. Consistent efforts have been made to develop gene therapy strategies using viral and non-viral vectors of gene delivery. Here, we have discussed both of these delivery methods and their properties. We have summarized the relative advantages and drawbacks of viral and non-viral vectors from the perspectives of safety, efficiency, and productivity. Recent developments such as clustered regularly interspaced short palindromic repeats (CRISPR)/Cas9-mediated gene editing and its use *in vivo* have been described here as well. Given recent advancements, gene therapy shows great promise to emerge as a next-generation therapeutic for many of the neurodevelopmental and neurodegenerative conditions.

## Introduction

The prevalence of neurological disorders has been rising across the globe and is often associated with an increasing socioeconomic burden. Many of these debilitating neurological conditions do not respond to conventional therapies. Therefore, it is imperative to explore novel treatment modalities like gene therapy. Recent groundbreaking gene therapy trials on patients with spinal muscular atrophy type 1 (SMA1) resulted in superior motor function and longer survival compared to the control group (Mendell et al., 2017). These investigations advocate for the further development of gene therapy strategies to treat neurological conditions by replacing the disease-causing mutant gene with a healthy copy or inactivating the malfunctioning disease-causing mutant gene. RNA interference (RNAi) technology, has been one of the popular approaches to inactivate target gene expression accomplished by a small non-coding RNA of varying length that binds a complementary sequence of the mRNA target (Davidson and Boudreau, 2007; Jagannath and Wood, 2007; Aguiar et al., 2017; Balwani et al., 2020). Besides these, studies have been conducted in recent years to edit the mutated copy of the gene itself *in vitro* and *in vivo.* Such an approach is known as gene editing (Li et al., 2020).

Clustered regularly interspaced short palindromic repeats (CRISPR)/Cas9 is one of the widely used gene editing tools. This method requires the delivery of Cas9 ribonucleoprotein (RNP) consisting of the Cas9 protein with guide RNA (gRNA) to the target cells. CRISPR/Cas9-mediated genome engineering is quite precise and straightforward due to the target specificity and simple design of gRNA. Studies employing CRISPR have already exhibited efficiency in preclinical models of neurological disorders (Gaj et al., 2017; Yang et al., 2017; György et al., 2018; Lee et al., 2018; Ekman et al., 2019; Ricci and Colasante, 2021). Moreover, a recent report suggests that CRISPR technology is in fact promising in clinical trials (Frangoul et al., 2021). While we expect CRISPR-based gene editing will soon change the therapeutic landscape of complex neurological disorders, the double-stranded break it creates in the host genome is the major drawback of Cas9-mediated editing. The non-homologous end joining (NHEJ) pathway is error-prone and often associated with undesired insertion or deletion (indel) mutations (Moore and Haber, 1996; Kosicki et al., 2018; Song et al., 2021). Further, non-dividing cells are incapable of undergoing homology-directed repair (HDR)-mediated editing which reduces the overall efficiency of these processes (Iyama and Wilson, 2013; Kantor et al., 2020). Base editing offers an alternative that could overcome these challenges given base editors do not rely on double-stranded DNA break (Komor et al., 2016; Eid et al., 2018; Anzalone et al., 2020). The two major base editors, cytosine base editors (CBEs) and adenine base editors (ABEs) can introduce all four transition mutations (A→G, G→A, C→T, T→C) (Gaudelli et al., 2017; Qi et al., 2020; Qin et al., 2020; Yu et al., 2020). In principle, ∼30% of all known human pathogenic single nucleotide polymorphisms (SNPs) can be targeted using base editors (Anzalone et al., 2020). Prime editors, the newest players in the league, are capable of introducing all four transitions, as well as all eight transversion mutations. Theoretically, prime editing can edit ∼89% of all known human pathogenic SNPs (Kantor et al., 2020). Hence, base editing and prime editing tools are perceived as extremely powerful strategies to target disease-causing point mutations. Such progress will be translated to patients and pave the way for gene therapy to be the next generation therapeutic by reverting the disease-causing mutation to the normal gene, a remarkable way to treat a disease.

Successful gene therapy strategies often rely on successful gene delivery methods. There are broadly two categories of gene delivery strategies; viral vector-mediated and nonviral vector-mediated gene delivery. However, apart from these two strategies, physical methods have also been tested as a gene delivery strategy. Nevertheless, due to a lack of target specificity, physical methods such as sonoporation are not widely accepted in the field. Here, we narrate the major gene delivery strategies and their relative advantages and disadvantages for brain targeting. Broadly, viral vectors are considered to be more efficient while non-viral vectors are on the whole less toxic and immunogenic. However, both delivery methods show exciting and hopeful recent advancements.

## Viral Vector-Mediated Gene Delivery

Viruses have been an alluring vector for gene therapy due to their efficiency infecting and delivering genetic material to cells. However, selecting an appropriate viral vector for gene therapy requires stringent parameters. Generally, there are three universally accepted criteria for a good gene delivery viral vector (Scheller and Krebsbach, 2009); 1. safe and non-immunogenic, 2. able to protect the transgene, and 3. capable of prolonged and tissue-specific transgene expression (if applicable). Considering these factors in mind, adenoviruses, lentiviruses, and adeno-associated viruses (AAVs) have been used to develop effective gene delivery vehicles targeting a wide range of neurological indications (Lapchak et al., 1997; Azzouz et al., 2002; Albert et al., 2017; Mendell et al., 2017). As viral vectors manifest a high gene delivery efficiency *in vivo*, they have received the limelight in the current decade for their use in gene therapy. Their applications are summarized in Table 1 and briefly described below:

**TABLE 1:** Gene therapy for neurological disorders using viral and non-viral vectors.

Delivery vehicles/vectors	Target diseases	Preclinical/Clinical stage	Target gene	Gene therapy approach	Outcome/Current status
**Viral vectors**					
Adenovirus (Ad)	PD	Preclinical (6-OHDA lesioned rats)	*GDNF*	Ad-mediated *hGDNF* expression	Improved locomotor behavior; increased DA and DOPAC levels in striatum and substantia nigra; shown to be neuroprotective (Lapchak et al., 1997; Choi-Lundberg et al., 1998)
	HD	Preclinical (Quinolinic acid-lesioned rats)	*BDNF*	Ad-mediated *BDNF* expression	Neuroprotection from quinolinic acid-induced neuronal death and reduced lesion volume (Bemelmans et al., 1999)
Lentivirus	AD	Preclinical (APP23 transgenic mice)	*PGC1α*	Lentiviral vector-mediated *hPGC1α* expression	Reduced Aβ deposition with improved spatial memory, decreased proinflammatory cytokine, and neuroprotection (Katsouri et al., 2016)
	PD	Preclinical (6-OHDA lesioned rats)	*TH, AADC, CH1*	Lentiviral vector-mediated *TH, AADC,* and *CH1* expression	Reduction of apomorphine-induced motor asymmetry and sustained catecholamine production (Azzouz et al., 2002)
	PD	Clinical [NCT01856439, NCT00627588]	*TH, AADC, CH1*	ProSavin (Lentiviral vector-mediated *TH, AADC, CH1* expression)	Phase I/II (ProSavin is safe and well tolerated in PD patients; moderate improvements in motor behavior reported) (Palfi et al., 2014; Palfi et al., 2018)
	Refractory focal epilepsy	Clinical [NCT04601974]	*KCNA1*	Lentiviral vector-mediated expression of engineered potassium channels in excitatory neurons	Phase I/IIa (Study ongoing)
Adeno-associated virus (AAV)	PD	Preclinical (MPTP lesioned rhesus macaques)	*GDNF*	AAV2-*GDNF* (AAV2-mediated *GDNF* delivery)	No histopathological and immune reaction and no loss of body weight (Su et al., 2009)
	PD	Clinical [NCT04167540]	*GDNF*	AAV2-*GDNF* (AAV2-mediated *GDNF* delivery)	Phase Ib (ongoing)
	PD	Clinical [NCT00195143]	*GAD*	AAV-*GAD* (AAV-mediated *GAD* delivery)	Phase I (completed); Patients tolerated the therapy with improvements in motor scores (Unified Parkinson’s Disease Rating Scale, UPDRS) (Kaplitt et al., 2007)
	PD	Clinical [NCT00229736]	*AADC*	AAV2-*hAADC* (AAV2-mediated *hAADC* delivery)	Phase I (completed); Patients tolerated the therapy and transgene expression sustained for 4 years (Mittermeyer et al., 2012)
	PD	Clinical [NCT01973543]	*AADC*	VY-AADC01 (AAV2-mediated *hAADC* delivery)	Phase I (completed); The therapy was well tolerated in patients; dose-dependent transgene expression and subsequent improvement in clinical outcome was observed (Christine et al., 2019)
	PD	Clinical [NCT00985517]	*NTN*	CERE-120 (AAV2-mediated *NTN* delivery)	Phase II (completed); Patients tolerated the therapy (Bartus et al., 2013)
	ALS	Clinical	*SOD1*	AAV-*miR-SOD1* (disrupting *SOD1* gene expression, AAVrh.10)	Phase I/II will be initiated (Mueller et al., 2020)
	ALS	Preclinical (G93A-SOD1 mouse model)	*SOD1*	AAV9-*SaCas9-hSOD1* (*in vivo* gene editing)	Improved motor function, reduced muscle atrophy, and increase in survivability (Gaj et al., 2017)
	ALS	Preclinical (G93A-SOD1 mouse model)	*SOD1*	AAV-mediated cytidine base editor (CBE) delivery	Longer survival and slow disease progression observed; improved neuromuscular functions; reduced levels of SOD1 immunoreactive inclusions seen (Lim et al., 2020)
	AD	Clinical [NCT05040217]	*BDNF*	AAV2-*BDNF* (AAV2-mediated *BDNF* expression)	Phase I (study ongoing)
	AD	Clinical [NCT04133454]	*hTERT*	AAV-*hTERT* (AAV-mediated telomerase expression)	Phase I (status unknown)
	AD	Clinical [NCT03634007]	*APOE2*	LX 1001; AAVrh.10hAPOE2 (AAV-mediated expression of *APOE2*)	Phase I (study ongoing)
	AD	Clinical [NCT00087789, NCT00876863]	*NGF*	Cere 110; AAV2-*NGF* (AAV2-mediated *NGF* expression)	Phase II (study completed); Cere 110 was safe and well tolerated but inefficient (Mandel, 2010)
	AD	Preclinical (Tg 2576 mice)	*APP*	AAV-mediated depletion of *APP* ^*SW*^ mutation (CRISPR gene editing)	Reduced level of Aβ secretion; *in vivo* indel formation (György et al., 2018)
	FTD	Clinical [NCT04747431]	*GRN*	PBFT02; AAV1-*GRN* (AAV1-mediated *GRN* expression)	Phase I/II (study ongoing)
	HD	Clinical [NCT04120493, NCT05243017]	*HTT*	AMT-130; rAAV5-*miHTT* (perturbing HTT expression)	Phase I/II (study ongoing) (Spronck et al., 2021)
	HD	Preclinical (R6/2 mice)	*HTT*	AAV1-*SaCas9-HTT* (CRISPR-mediated gene editing disrupting HTT expression)	Reduced mHTT level and associated inclusion bodies; increased survival (Ekman et al., 2019)
	HD	Preclinical (HD140Q-KI mice)	*HTT*	AAV-*HTT-gRNA/AAV-CMV-Cas9* with ratio 1:4 (CRISPR-mediated gene editing disrupting *HTT* expression)	Reduced mHTT level; improved motor functions (Yang et al., 2017)
	RTT	Preclinical (Mecp2−/ymice)	*MECP2*	AAV-*Mecp2* (AAV-mediated *Mecp2* expression)	Systemic administration led to liver toxicity; cerebroventricular administration resulted in improved survival and alleviated RTT like aggregate severity score (Gadalla et al., 2017)
	SMA	Clinical [NCT03306277, NCT02122952]	*SMN1*	Zolgensma (AAV9-*CMV-SMN1*; gene replacement therapy)	Phase III (study completed); Safe, well tolerated, approved for use (Mendell et al., 2017)
	Giant axonal neuropathy	Clinical [NCT02362438]	*GAN*	scAAV9/JeT-*GAN* (gene transfer therapy)	Phase I (study ongoing)
	Friedreich’s ataxia	Clinical [NCT05302271]	*FXN*	AAVrh.*10hFXN*; (gene transfer therapy)	Phase I (study ongoing)
	Niemann-Pick disease type C	Preclinical (*Npc1^tm(I1061T)Dso^* mice)	*Npc1*	AAV-CBE; AAV9-mediated delivery of cytosine base editor	Modest increase in lifespan of the mice following correction of disease-causing mutation (Levy et al., 2020)
**Non-viral vectors**					
Polymer-based vectors	PD	Preclinical (6-OHDA lesioned rats)	*VEGF*	PEI-PLL mediated *VEGF* gene delivery	Prevented loss of motor functions; protected loss of dopaminergic neurons of SNpc; prevented microglial activation and apoptosis (Sheikh et al., 2017)
	PD	Preclinical (6-OHDA lesioned rats)	*hGDNF*	Lactoferrin modified PAMAM dendrimer mediated *GDNF* gene delivery	Improved motor behavior; decreased loss of dopaminergic neurons; increased monoamine neurotransmitter levels (Huang et al., 2009)
	PD	Preclinical (Rotenone-lesioned PD rats)	*hGDNF*	Lactoferrin modified PAMAM dendrimer mediated *GDNF* gene delivery	Improved motor behavior; decreased loss of dopaminergic neurons; increased monoamine neurotransmitter levels (Huang et al., 2010)
	AD	BALB/c mice	*Bace1*	Rabies virus glycoprotein (RVG)-modified poly(mannitol-co-PEI) gene transporter (PMT)-mediated *Bace1* siRNA delivery	BACE1 protein and mRNA level reduced in the hippocampus and cortex; accompanied by reduced Aβ42 level (Park et al., 2015)
Lipid-based vectors	AD	Preclinical (C57BL/6 mice)	*APOE2*	Transferrin-Penetratin modified liposomes for delivery of *ApoE2*	Increased expression of apolipoprotein E2 in the brain (Dos Santos Rodrigues et al., 2019)
	PD	Preclinical (6-OHDA lesioned rats)	*TH*	*TH plasmid* in PEGylated immunoliposome (PIL) targeted *via* rat transferrin receptor (TfR)	Increased TH level in the striatum; ameliorated apomorphine-induced rotational behavior (Zhang et al., 2003; Zhang et al., 2004; Pardridge, 2005)
	PD	Preclinical (6-OHDA lesioned rats)	*GDNF*	PEGylated liposome-microbubble-mediated delivery of *GDNF* plasmid	Increased GDNF expression (mRNA and protein); averted 6-OHDA-induced drop of TH and DAT level; prevented the apomorphine-induced rotational behavior (Yue et al., 2018)
	AD	Preclinical (APP/PS1 transgenic mice)	*BDNF*	Liposomal nanoparticle-mediated *BDNF* gene delivery	Two-fold increase in BDNF level with concomitant reduction (>40%) of Aβ peptide; Plaque load was reduced with subsequent increase in synaptic proteins like Synaptophysin, and PSD-95 (Arora et al., 2022)
Nanoparticle-based vectors	PD	Preclinical (MPTP injected mice)	*SNCA*	Superparamagnetic nanoparticle (Fe_3_O_4_ nanoparticle)-mediated delivery of shRNA for *SNCA*	Reduced α-synuclein and concomitant increase of TH level in substantia nigra; improved motor function (longer distance travelled in open field arena) (Niu et al., 2017)
	PD	Preclinical (MPTP injected mice)	*SNCA*	Gold nanoparticle-mediated silencing of *SNCA* expression (using RNAi technology)	SNCA level was suppressed; reduced damage of nigrostriatal pathway (based on Nissl staining) (Hu et al., 2018)
	PD	Preclinical (MPTP injected mice)	*SNCA*	Gold nanoparticle-mediated silencing of *SNCA* expression (using RNAi technology)	Elevated TH level; reduced α-synuclein aggregate in substantia nigra; improved motor function; ameliorated LTP deficit (Liu et al., 2020)
	AD	Preclinical (5XFAD transgenic mice)	*Bace1*	R7L10 peptide (nanocomplex)-mediated Cas9 RNP delivery targeting *Bace1* (CRISPR gene editing)	Reduction in BACE1 expression; decreased Aβ plaque formation; associative learning and spatial working memory rescued (Park et al., 2019)
	FXS	Preclinical (Fmr1 knockout mice)	*Grm5*	CRISPR-Gold -mediated delivery of Cas9 RNP to knockout *Grm5*	Reduced mGluR5 level in the striatum; rescued repetitive behavior (Lee et al., 2018)

The table depicts major preclinical and clinical studies to treat neurological disorders employing gene therapy modalities. Abbreviations (PD, Parkinson’s disease; HD, Huntington’s disease; AD, Alzheimer’s disease; ALS, Amyotrophic lateral sclerosis; FTD, Frontotemporal dementia; RTT, Rett syndrome; SMA, Spinal muscular atrophy; FXS, Fragile X syndrome; GDNF, Glial derived neurotropic factor; BDNF, Brain derived neurotropic factor; PGC1α, Peroxisome proliferator-activated receptor gamma coactivator 1-alpha; TH, Tyrosine hydroxylase; AADC, Aromatic amino acid dopa decarboxylase; CH1, GTP cyclohydrolase 1; KCNA1, Voltage gated potassium channel Kv1.1; GAD, Glutamic acid decarboxylase; NTN, Neurturin; SOD1, Superoxide dismutase; TERT, Telomerase reverse transcriptase; APOE2, Apolipoprotein E2; NGF, Nerve growth factor; APP, Amyloid precursor protein; GRN, Progranulin; HTT, Huntingtin; MECP2, Methyl-CpG Binding Protein 2; SMN1, Survival motor neuron 1; GAN, Gigaxonin; FXN, Frataxin; Npc1, NPC intracellular cholesterol transporter 1; VEGF, Vascular endothelial growth factor; BACE1, β-Secretase 1; SNCA, α-Synuclein; Grm5, Metabotropic glutamate receptor 5).

**Adenovirus-based vectors**: Adenoviral vectors can package 5–10 kb of naked double-stranded DNA (dsDNA) (He et al., 1998). They can infect quite a broad range of cells and express episomally (Leblois et al., 2000; Kreppel and Kochanek, 2004; Shu et al., 2016). Two of the major drawbacks of the adenoviral vectors have been their immunogenicity and low efficiency to cross blood-brain-barrier (BBB) (Kafri et al., 1998; Franklin et al., 1999; Tang et al., 2007; Lundstrom, 2018). Recently, efforts were made to utilize the transcellular transport pathway to facilitate the BBB penetration of adenovirus serotype-5 (Ad5) vectors. Re-direction of Ad5 vectors to melanotransferrin transcytosis system promoted Ad5-mediated gene delivery through BBB (Tang et al., 2007).

**Lentivirus-based vectors**: Lentiviral vectors are categorized under the retroviruses and are capable of infecting both dividing and non-dividing cells (Naldini et al., 1996; Blömer et al., 1997; Yang et al., 2008; Lundstrom, 2018). These vectors can package up to 10 kb of single-stranded RNA (ssRNA) manifesting long-term transgene expression (Sakuma et al., 2012; Lundstrom, 2018; Kalidasan et al., 2021). One key concern for the use of lentiviral vectors has been the possibility of their integration into the host genome leading to insertional mutagenesis. Integration-defective lentiviral vectors have been developed to circumvent the possibilities of insertional mutagenesis in the brain (Philippe et al., 2006).

**AAV-based vectors**: AAV-based vectors generally carry single-stranded DNA (ssDNA) and can package ∼4.8 kb of content (Carvalho et al., 2017; Lundstrom, 2018). Their ability to infect a wide range of dividing and non-dividing cells makes them suitable for many therapeutic trials. The existence of more than 12 serotypes and their various features have made AAV-based vectors the most attractive vehicle (Ingusci et al., 2019). A number of natural AAV serotypes exhibit both anterograde and retrograde trafficking while natural serotypes such as AAV1, AAV2, AAV6, and AAV9 require high vector doses for retrograde trafficking due to the relative inefficiency (Haery et al., 2019). AAV1, AAV5, AAV8, and AAV9 transduce neurons as well as astrocytes and oligodendrocytes while AAV2 transduce mostly neurons (Tenenbaum et al., 2004; Haery et al., 2019). Compared to other serotypes, AAV9 manifests superior BBB crossing ability when assessed in neonatal mouse CNS (Foust et al., 2009; Zhang et al., 2011; Haery et al., 2019). Various AAV serotypes can either exist as an episome or can integrate into the host genome. AAV vectors are often immunogenic, however, the use of different serotypes for subsequent administrations has shown promise in combating this (Lundstrom, 2018).

### Advantages of Using Viral Gene Delivery

In recent days, the AAV vectors have gained a lot of attention for gene therapy given their broad range of cell tropism and suitability for subsequent engineering (Srivastava, 2016; Li and Samulski, 2020). Recombinant AAV vectors have unique features such as their capability to transport through extracellular space due to the small particle size. Furthermore, replication-incompetent AAV vectors are often considered as one of the safer options for *in vivo* use (Hudry and Vandenberghe, 2019). They can elicit a stable and sustained transgene expression as an episome. Recently, the field has seen enormous progress in developing AAV-mediated targeted gene therapy approaches in the CNS (see Table 1).

### Disadvantages of Using Viral Gene Delivery

Despite having numerous advantages, AAV-mediated gene delivery requires further fine-tuning. Some of the critical challenges include identifying a safe and less-invasive route of administration, overcoming large-scale manufacturing obstacles, and successful translation from preclinical models to humans (complicated by immunological differences and presence of BBB). Besides these, another key challenge has been the delivery of larger cargos. Recent advancements in gene editing technology require the delivery of massive machinery like CRISPR/Cas9 to target cells. However, considering the limited carrying capacity of AAV, the cargo would have to be split into two or more vectors (Trapani, 2019; Akil, 2020). Such an approach reduces transduction efficiency as the chances of simultaneous reach of multiple vectors to the target cells become stochastic. Therefore, it is important to have a vehicle that can carry larger complexes to target cells.

## Non-Viral Vector-Mediated Gene Delivery

Non-viral vectors have been used as a vehicle for many different gene therapy trials even though the system has not gotten the same fame seen with the viral vector models. Nevertheless, non-viral vectors have shown promise in many of the aspects of an ideal gene therapy vector. Broadly, there are three major strategies of non-viral vector-mediated gene therapy such as polymer-based vectors, lipid-based vectors, and nanoparticle-based vectors (Mintzer and Simanek, 2009; Jayant et al., 2016; Salameh et al., 2020). See Table 1 for their applications.

**Polymer-based vectors**: Polyethyleneimine (PEI) and poly-L-Lysine (PLL) are cationic polymers which are popularly tested *in vivo* CNS for gene therapy (Boussif et al., 1995; Mousazadeh et al., 2007; Mintzer and Simanek, 2009; Sheikh et al., 2017; Zheng et al., 2021). Each polymer-based vector demonstrates distinct features; PEI is versatile given it can be designed to be different lengths, be branched or linear, undergo functional group substitution or addition (Morille et al., 2008; Jayant et al., 2016); PLL is unique due to its biodegradable nature, which is advantage for *in vivo* use (Morille et al., 2008; Jayant et al., 2016). Studies demonstrated that cytotoxicity of PEI and PLL is directly related to molecular weight and pK_a_, with higher molecular weight and more cationic materials being more toxic (Mintzer and Simanek, 2009; Monnery et al., 2017). Further investigation showed PLL conjugated with apoprotein E (apoE)-derived peptide demonstrated to cross BBB (Mousazadeh et al., 2007). Dendrimers, highly branched spherical polymers, have also been widely investigated as a gene delivery vehicle. Polyamidoamine (PAMAM) is the most common form of a dendrimer due to its ample transfection efficiency (Wang et al., 2011; Zhu et al., 2019; Mignani et al., 2021). Cytotoxicity of dendrimers as a result of their surface charge and chemical structure was shown to be alleviated by polyethylene glycol (PEG) modification (PEGylation) (Jayant et al., 2016). Additionally, a single intranasal administration of PAMAM dendrimers was shown to modulate brain-derived neurotrophic factor gene expression in the brain (Win-Shwe et al., 2014).

**Lipid-based vectors**: Liposomes consist of spherical concentric bilipid layers capable of carrying nucleic acid to target cells (Gao et al., 2013). Liposomes have been widely used as non-viral vectors for CNS targeting (Zhang et al., 2003; Zhang et al., 2004; Dos Santos Rodrigues et al., 2019). However, simple liposomes face multiple obstacles including lysosome-mediated degradation and reduced nuclear uptake. Cationic liposomes are comparatively more efficient in transfecting larger nucleic acids and are easy to handle, though, they tend to form an aggregate in biological fluids (Jayant et al., 2016; Ewert et al., 2021). Niosomes are more stable than liposomes (Bartelds et al., 2018; Ge et al., 2019). The major limitation of niosomes has been the possible aggregation, fusion, and leaking, which could be circumvented by the usage of proniosomes (Hu and Rhodes, 2000). Further, PEGylation of niosomes led to improved gene delivery, reduced interaction with plasma proteins, and prohibited aggregation in the serum (Huang et al., 2008). To foster the BBB crossing, trojan horse liposomes (THL) has been developed. THL relies on its monoclonal antibody component to bind with the cognate receptors (e.g., transferrin receptors or insulin receptors) present on BBB or the cell surface (Boado and Pardridge, 2011). Another approach is designing dual-functionalized liposomes having penetratin and transferrin attached to the surface (Dos Santos Rodrigues et al., 2018; Dos Santos Rodrigues et al., 2019).

**Nanoparticle-based vectors**: Nanoparticle-based vectors are at the forefront of gene delivery modalities as a result of their safety profile as well as their cost-effective production method. Nanoparticles are defined as solid colloidal particles with sizes ranging from 1 to 1000 nm consisting of macromolecular materials in which the active compound (drug/biologically active material e.g., DNA/RNA/protein) is encapsulated, absorbed, or entrapped (Kreuter, 2014). Generally, nanoparticles are biocompatible and readily biodegradable, making them suitable gene delivery vehicles for CNS targeting (Calzoni et al., 2019). Major components of nanoparticles are poly-butylcyanoacrylide (PBCA), poly-lactic acid (PLA) and related copolymers (Kreuter, 2014). In many cases, modulating the surface properties of these nanoparticles by PEGylation, or polysorbate-80 coating improves gene delivery efficiency. PEGylation, in particular, prolongs systemic circulation time and reduces the immunogenicity of the nanoparticles (Suk et al., 2016). Whereas, polysorbate-80 coating was shown to enhance BBB crossing (Ren et al., 2009). Similar to liposomes, functionalizing nanoparticles with specific ligands shown to be capable to cross BBB (Lombardo et al., 2020). For example, ApoE-modified nanoparticles have been shown to circumvent BBB (Wagner et al., 2012). Other investigations demonstrated that insulin-targeted gold nanoparticles can effectively cross BBB *via* receptor-mediated endocytosis (Shilo et al., 2014). Gold nanoparticle was also shown to deliver the large cargo such as CRISPR/Cas9 RNP leading to a behavioral rescue in a preclinical mouse model of fragile X syndrome (FXS) without showing any significant cytotoxicity (Horejs, 2018; Lee et al., 2018; Trenkmann, 2018).

### Advantages of Using Non-Viral Gene Delivery

Non-viral vectors have multiple advantages over viral vectors. Lower toxicity and immunogenicity make non-viral vectors a safer tool for gene delivery. Other advantages of the non-viral vectors would be the ease of production, thereby, cost-effectiveness, and their ability to be engineered (Ramamoorth and Narvekar, 2015). On top of that, non-viral vectors can transfer bigger sizes of nucleic acid residues and/or proteins (Morrell et al., 2008; Chang et al., 2017; Lee et al., 2018; Park et al., 2019; Zhang et al., 2021; Jubair et al., 2021; O'Keeffe Ahern et al., 2022). Considering the current progress in gene editing technology, transferring larger biomolecule complexes is desired.

### Disadvantages of Using Non-Viral Gene Delivery

Regardless of many advantages over the viral vectors, non-viral vectors do face some challenges. Firstly, the comprehensive mechanisms of their actions are not well elucidated (Wu et al., 2018). Hence, it has been difficult to strike the optimum balance between efficiency and toxicity. Secondly, non-viral vectors generally exhibit lower efficiency for CNS targeting compared to viral vectors (Mintzer and Simanek, 2009; Jayant et al., 2016). However, recent advancements in chemical modifications of the lipid carriers and the other non-viral vectors are promising in enhancing CNS targeting. Another hurdle has been ‘protein corona’ formation (Corbo et al., 2016). Systemically administered lipid nanoparticles encounter and interact with countless biomolecules which change their surface properties and form protein coronas (Caracciolo, 2015; Giulimondi et al., 2019). This phenomenon can impact cellular uptake, biodistribution, immune reaction, and toxicity of the vector (Corbo et al., 2016). Therefore, it is imperative to develop a safe yet efficient delivery modality.

## Discussion

Gene therapy for complex neurological conditions including neurodevelopmental and neurodegenerative disorders is still in its adolescence. The field has encountered a lot of challenges in the last couple of decades. One of the major challenges has been safety and tolerability. Subsequent efforts and advancements have led to many successful clinical trials addressing the safety concern. Nevertheless, we have seen a surge in the number of clinical trials targeting complex disorders employing gene therapy approaches in the last couple of decades (Kaplitt et al., 2007; Mittermeyer et al., 2012; Palfi et al., 2014; Mendell et al., 2017; Palfi et al., 2018; Christine et al., 2019). It is noteworthy to mention that the proportion of these gene therapy trials is still not on par with conventional small-molecule therapeutic development. Therefore, it is critical to reiterate that more efforts are needed for the successful translation of gene therapy strategies from bench to bedside. Gene therapy could be a game-changer in treating rare monogenic disorders in the years to come. Debilitating indications like amyotrophic lateral sclerosis (ALS) and Huntington’s disease (HD) are quite aggressive and lack an effective disease-modifying therapy. In most cases, the only treatment available is symptomatic alleviation. In such a scenario, targeting the underlying etiology could be both beneficial and advantageous. The development of gene therapy strategies has shown us a light at the end of the tunnel.

**FIGURE 1:**
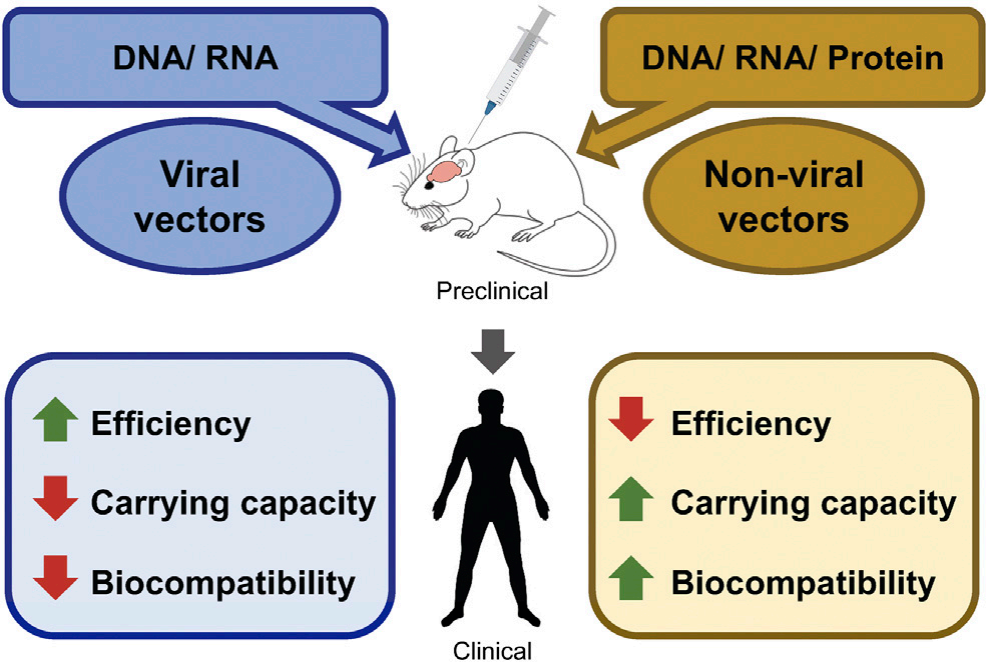
Perspectives of advantages/disadvantages of viral-, and non-viral gene delivery methods. Schematic diagram shows the possible packaging materials of viral and non-viral vectors. Packaged vectors can be tested in the preclinical models of various neurological disorders. Followed by successful preclinical trials, human clinical trials can be conducted. The advantages and the disadvantages of each vector are marked in green and red, respectively.

Successful gene delivery strategies are often the rate-limiting step for efficient gene therapy (see Figure 1). Viral vectors have been designed to evade the immune system and deliver their nucleic acids to target organs (Chan et al., 2021). Such a function constitutes one of the mechanisms of increased efficiency of viral vector-mediated gene delivery. The American Society of Gene & Cell Therapy (ASGCT) proclaimed in the Q1-2021 quarterly data report that 89% of the total gene therapies in development employ viral vectors including AAV (42%), lentivirus (29.9%), and adenovirus (12.6%). A similar trend is expected for the gene therapy landscape of neurological disorders. Considering current gene therapy preclinical development, neurological disorders are the second most widely targeted non-cancer non-rare indications. Furthermore, Zolgensma, the only FDA-approved gene therapy against SMA utilizes the AAV9 vector, indicating that viral vectors have been the primary choice (Al-Zaidy et al., 2019; Urquhart, 2019).

Gene delivery strategies are rapidly evolving to deliver a wide assortment of bioactive materials such as nucleic acids, proteins, or gene editing tools into the brain. However, bioactive materials (e.g., nucleic acids) are susceptible to degradation by serum nucleases and need protective modifications for successful delivery to the cells (Chiou et al., 1994; Juliano, 2016; Roberts et al., 2020). Therefore, current progress towards designing non-viral vectors which can protect bioactive materials against degradation inside the body becomes promising. Gene delivery through PEGylated liposomes can effectively protect nucleic acids while retaining their bioavailability (Suk et al., 2016). In addition, toxicity has been one of the major concerns masking the flare of gene therapy strategies. A good number of efforts have been made to design biocompatible and biodegradable cargo for safe gene delivery (Han et al., 2000). Non-viral vectors have shown an upper hand when it comes to safe gene delivery to the target tissues. Studies are ongoing to make non-viral vectors more efficient above and beyond being safe. Current advancements in gene editing technology also contribute to the popularization of non-viral vectors.

Lastly, most of the efficient viral vectors (AAV-mediated vectors) have limitations in their carrying capacity, whereas, non-viral vectors can carry larger DNA/RNA as well as proteins quite effortlessly (see Figure 1). This makes them suitable to deliver gene editing machinery such as CRISPR/Cas9. The development of virus-like particles (VLP) which are composed of virus assembly proteins, but lack the viral genetic material is one of the recent advancements in the field to overcome the limitations of both types of vectors. VLPs have become an attractive vehicle as they possess a similar efficiency to viral vectors without having the associated risk of genomic integration of viral gene construct. VLP-mediated gene editing was successful in *ex vivo* human cells as well as *in vivo* mouse brains (Banskota et al., 2022). With these results, VLP could turn out to be an efficient gene therapy vector in the future.

In conclusion, gene therapy is undoubtedly one of the crucial developments of this century. No wonder the Nobel prize 2020 (Chemistry) was conferred to Drs. Jennifer Doudna and Emmanuelle Charpentier for their discoveries in CRISPR/Cas9-mediated gene editing. The cutting-edge technological advancements in gene therapy are giving hope to millions of people suffering from excruciating neurological conditions. Despite great progress in the field, we are still dealing with challenges in bringing gene therapy medicines to market. Nevertheless, the consistent efforts and developments by experts across the globe are encouraging. With the increase in cross-functional collaborations in these sectors, we can expect to see various approved gene therapy treatments for patients in the near future. Gene therapy will emerge as next-generation therapeutics for many neurodevelopmental and neurodegenerative diseases in the decades to come.
